# Accuracy of a Smartphone Application Measuring Snoring in Adults—How Smart Is It Actually?

**DOI:** 10.3390/ijerph18147326

**Published:** 2021-07-08

**Authors:** Katharina Klaus, Anna-Lena Stummer, Sabine Ruf

**Affiliations:** 1 Department of Orthodontics, Justus-Liebig University Giessen, 35392 Giessen, Germany; anna.l.stummer@dentist.med.uni-giessen.de (A.-L.S.); sabine.ruf@dentist.med.uni-giessen.de (S.R.); 2 Department of Oral and Maxillofacial Surgery, Diakonie Klinikum Jung-Stilling Siegen, 57074 Siegen, Germany

**Keywords:** application, app, eHealth, mHealth, obstructive sleep apnea, OSA, smartphone, snoring

## Abstract

About 40% of the adult population is affected by snoring, which is closely related to obstructive sleep apnea (OSA) and can be associated with serious health implications. Commercial smartphone applications (apps) offer the possibility of monitoring snoring at home. However, the number of validation studies addressing snoring apps is limited. The purpose of the present study was to assess the accuracy of recorded snoring using the free version of the app SnoreLab (Reviva Softworks Ltd., London, UK) in comparison to a full-night polygraphic measurement (Miniscreen plus, Löwenstein Medical GmbH & Co., KG, Bad Ems, Germany). Nineteen healthy adult volunteers (4 female, 15 male, mean age: 38.9 ± 19.4 years) underwent simultaneous polygraphic and SnoreLab app measurement for one night at home. Parameters obtained by the SnoreLab app were: starting/ending time of monitoring, time in bed, duration and percent of quiet sleep, light, loud and epic snoring, total snoring time and Snore Score, a specific score obtained by the SnoreLab app. Data obtained from polygraphy were: starting/ending time of monitoring, time in bed, total snoring time, snore index (SI), snore index obstructive (SI obstructive) and apnea-hypopnea-index (AHI). For different thresholds of percentage snoring per night, accuracy, sensitivity, specificity, positive and negative predictive values were calculated. Comparison of methods was undertaken by Spearman-Rho correlations and Bland-Altman plots. The SnoreLab app provides acceptable accuracy values measuring snoring >50% per night: 94.7% accuracy, 100% sensitivity, 94.1% specificity, 66.6% positive prediction value and 100% negative prediction value. Best agreement between both methods was achieved in comparing the sum of loud and epic snoring ratios obtained by the SnoreLab app with the total snoring ratio measured by polygraphy. Obstructive events could not be detected by the SnoreLab app. Compared to polygraphy, the SnoreLab app provides acceptable accuracy values regarding the measurement of especially heavy snoring.

## 1. Introduction

Snoring is an age- and gender-dependent phenomenon and affects 40% of the adult population [1,2]. According to the International Classification of Sleep Disorders (ICSD-3), snoring is ranked among the sleep-related breathing disorders [3] and frequently acts as a symptom of obstructive sleep apnea (OSA) or the upper airway resistance syndrome (UARS) [2,4,5]. OSA has been associated with serious health implications like daytime sleepiness and increased risk of accidents, pulmonary hypertension, heart failure, stroke, diabetes mellitus and depression. However, the prove of causality for most of the comorbidities is lacking [6,7,8]. Nevertheless, even non-apneic snoring is reported to be associated with daytime sleepiness, hypertension and carotid atherosclerosis [2]. Furthermore, sleep-related disorders cause high economic costs: In Australia alone, inadequate sleep represented 4.6% of the national burden of disease [9]. 

Given the fact that OSA is commonly underdiagnosed and most patients with critical symptoms do not discuss them with their primary care physician [10,11], a screening of patients for snoring seems reasonable. The American Academy of Sleep Medicine recommends a screening for patients with obesity, family history of OSA, mandibular retrognathia and other known OSA comorbidities [5]. Mandibular retrognathia is diagnosed and treated by dentists and orthodontists. In mild to moderate OSA cases, when continuous positive airway pressure (CPAP) therapy is not well tolerated by the patient, manufacturing and insertion of a mandibular advancement appliance for night-time wear is recommended [12,13]. In cases of mandibular retrognathia with reduced posterior airway space and unsuccessful treatment approaches using CPAP and mandibular advancement devices, even an orthodontic/orthognathic treatment can be performed [14]. Orthodontists are also experienced in diagnosis and treatment of other dental and orofacial dysgnathias going along with disturbed breathing patterns and orofacial dysfunctions such as dental and skeletal open bite cases. Therefore, primary care physicians as well as dentists, respectively, orthodontists, should be aware of snoring patients for further referral to specialists in sleep medicine. 

In-laboratory polysomnography (PSG) remains the gold standard for the diagnosis of OSA [15,16], however, PSGs are not suitable for screening purposes due to their expensiveness and limited availability [17,18]. Therefore, the use of home sleep apnea testing with technically adequate tools like multi-channel polygraphy (PG) devices is recommended for the diagnosis of OSA in uncomplicated adult patients [16].

During the last few decades, the use of smartphones has become an integral part of daily life and users can select from 325,000 health-related apps in the common mobile app stores [19]. Regarding sleep, a variety of apps offer sleep tracking, sleep analysis or assessment of snoring. Pertinent reviews in the field state that scientific validation of commercial snoring apps is poor [17,20,21]. Especially snoring apps, using solely the smartphone without any secondary devices, such as external pulse oximeters, have only been validated in clinical setups based on one, respectively, two patients [22,23]. Another validation study [24] used a paid smartphone application and included 11 patients with snoring problems wearing an oral appliance at night, which could have influenced snoring during the investigation. Two research groups [25,26] described their development of audio-based snoring apps and validated them in 40 [25] and 120 [26] subjects, respectively. Nevertheless, both apps are not available in the common mobile app stores until now. The present study aimed at assessing the accuracy of a commonly used, commercially available free snoring app. Hence, a search with the term “snoring app” in the Google Play store as well as the Apple App store was undertaken. The app of the result list which had the highest number of downloads was chosen for the present study. 

Therefore, the purpose of the present study was to assess the accuracy of recorded snoring using the free version of the app SnoreLab (Reviva Softworks Ltd., London, UK; https://www.snorelab.com/; accessed on 24 June 2021) in comparison to a full-night polygraphic measurement (Miniscreen plus, Löwenstein Medical GmbH & Co., KG, Bad Ems, Germany; https://hul.de/en/produkt/mini-screen-plus-2/; accessed on 24 June 2021). 

## 2. Materials and Methods

The present explorative investigation was approved by the local ethics committee of the medical faculty of the Justus-Liebig-University Giessen (number 268/19). The study sample comprised 19 healthy, adult volunteers (5 female, 14 male) with a mean age of 38.9 years (standard deviation (SD) 19.4 years, age range 21 to 80 years). The mean Body Mass Index (BMI) was 26.9 (SD 4.7). Due to the fact that being overweight, respectively, obesity, is a main risk factor for OSA [6,7], the present population showed only a slightly increased BMI. Given the fact that the majority of the present study population is male, the BMI of the study population corresponds to the mean BMI in Germany (males aged 35–40 years: 26.4, females aged 35–40 years: 24.3, both sexes: 25.5) [27]. All volunteers reported occasional snoring, did not wear any kind of oral appliances at night which might have had influenced the breathing pattern and were recruited from the social environment of the university department staff. 

All subjects underwent one full-night polygraphic measurement of sleep at home using a ten-channel portable device (Miniscreen plus, Löwenstein Medical GmbH & Co., KG, Bad Ems, Germany; https://hul.de/en/produkt/mini-screen-plus-2/; accessed on 24 June 2021) with simultaneous snoring app recordings using the SnoreLab application (Reviva Softworks Ltd., London, UK; https://www.snorelab.com/; accessed on 24 June 2021) on their individual smartphone. For both the polygraphic measurement device as well as the snoring app, oral and written step-by-step instructions were provided. To avoid recording bias, subjects were advised to place their smartphone in a range of one to two meters near the pillow with the microphone of the phone directed towards their head. For the whole night, the smartphone was connected to a power source and should be set into flight mode. Furthermore, all volunteers were asked to keep windows closed and not to share their bedroom with a bedpartner or a pet. 

The following data were obtained from the SnoreLab app (SL):

- starting and ending time of monitoring
- time in bed
- monitoring time
- duration and ratio of quiet sleep, light, loud and epic snoring
- total time of snoring
- Snore Score, which is a specific score given by the SnoreLab app.

Data obtained from polygraphy (PG) were:

- starting and ending of monitoring
- time in bed
- total time of snoring
- snore index (SI, number of snoring events per hour)
- snore index obstructive (SI obstructive, number of snoring events per hour associated with obtrusive events)
- apnea-hypopnea-index (AHI) [3,6,7].

SnoreLab gives the opportunity to select manually how long it usually takes the user to fall asleep. This would explain some differences between the recorded time in bed and the monitoring time. Nevertheless, in the present patient sample, major discrepancies occurred between the recorded time in bed, the monitoring time and the summed-up times of the stages (quiet sleep; light, loud and epic snoring). In five cases, the summed-up times exceeded the recorded monitoring time by more than ten minutes and in one of the five by nearly one hour. To overcome this discrepancy, the times of the recorded stages (quiet sleep; light, loud and epic snoring) were summed up and used as the SnoreLab app monitoring time for further evaluation. For SnoreLab as well as polygraphy, percentage snoring was calculated as the ratio between snoring time and monitoring time. 

Statistical analysis was performed using IBM SPSS Statistics for Windows [28], version 25 (IBM Corporation, Armonk, NY, USA). Besides descriptive statistics; sensitivity, specificity, accuracy as well as positive and negative predictive values were calculated. Therefore, different minimal arbitrary thresholds for the percentage of total snoring per night recorded by polygraphy and the SnoreLab app were set. The number of results beyond and below the respective threshold was calculated in a cross table (Table 1). 

The different metrics were calculated as follows:

- Sensitivity = TP/(TP + FN)
- Specificity = TN/(FP + TN)
- Positive predictive value = TP/(TP + FP)
- Negative predictive value = TN/(FN + TN)
- Prevalence = TP/Total study population
- Accuracy = (TP + TN)/Total study population.

Due to the non-parametric distribution of data (Shapiro–Wilk test) [29], Spearman-Rho correlations were calculated and classified according to Hinkle et al. [30]. Additionally, the agreement between the two methods was analyzed by Bland–Altman plots [31,32]. As no sample size calculation was undertaken a priori, a post hoc power test was carried out using G*Power [33,34], Version 3.1 (HHU Düsseldorf, Düsseldorf, Germany). 

## 3. Results

**Figure 1:**
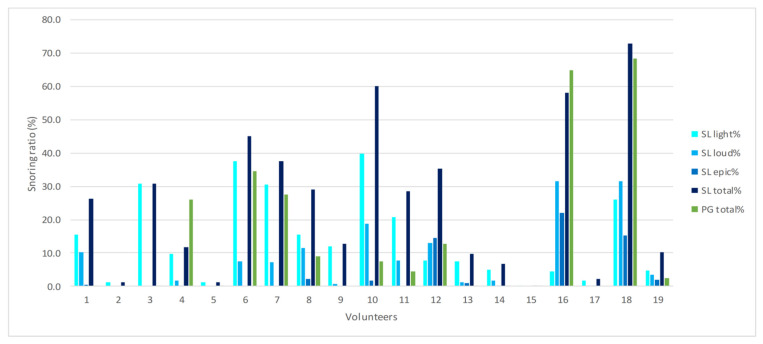
Individual percentage of snoring (%) measured by the SnoreLab app (SL) and polygraphy (PG) given for all 19 volunteers.

The measured snoring values for all 19 volunteers are given in Figure 1. In the majority of patients, the SnoreLab app recorded a higher total snoring ratio (dark blue bars) than polygraphy (green bars), while in only two individuals (4 and 16), polygraphy measured a higher percentage of snoring than SnoreLab. 

Analyzing the individual data by means of descriptive statistics, the total snoring ratio also reflected the tendency for an overrating of snoring by the SnoreLab app. Median values of percentage snoring per night showed a marked difference in the total snoring ratio (SL: 26.3%, PG: 2.4%), while the summed-up SnoreLab stages of loud and epic snoring (5.4%) approximated to the total polygraphic values (Table 2). Despite the non-normative distribution of data, no skewed distribution pattern was seen. Thus, the mean values and standard deviations are also given in Table 2. The mean values described a decreased difference between the total snoring ratios compared to the median values (SL: 25.3%, PG: 13.1%), but still underline the overestimation of snoring by the SnoreLab app. In concordance with the medians, the summed-up stages of loud and epic snoring recorded by the SnoreLab app (10.9%) approximated to the total polygraphic values.

Setting different arbitrary minimal thresholds for the percentage of total snoring per night (>5%, >10%, >25%, >50%) recorded by the SnoreLab app, sensitivity, specificity, accuracy, positive and negative predictive values in comparison to the gold standard polygraphy were highest with a threshold of >50% (Table 3). However, even for lower cutoff values, high sensitivities ranging from 80.0% to 100.0% and negative predictive values ranging from 88.8% to 100.0% were obtained, while specificities and positive predictive values decreased to about 50% and accuracies ranged between 63.2% and 73.6%. 

Despite the duration dependent methodological differences described above, the percentage of total snoring assessed by SnoreLab was highly correlated with the snoring ratio measured by polygraphy (r = 0.754). The same was true for the summed-up percentages of loud and epic snoring recorded by the SnoreLab app (r = 0.780). The post-hoc power analysis revealed that the correlation regarding the percentage of total snoring (r = 0.754), given an alpha-error of 0.05, had a power of 55.1% and was thus underpowered. The same was true for the correlation regarding the summed-up percentages of loud and epic snoring (r = 0.780), which resulted in a power of 65.2%.

**Figure 2:**
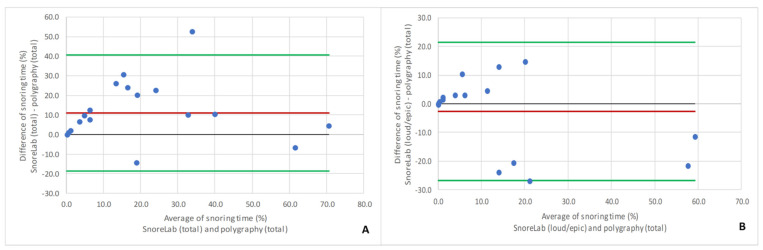
Bland–Altman plots for comparison of total snoring ratio measured by polygraphy with (**A**) total snoring ratio and (**B**) loud + epic snoring ratio measured by the SnoreLab app (**B**). Red line: mean difference of both methods; black line: optimal agreement between the methods; green lines: 95% limits of agreement.

The Bland–Altman analysis showed that the mean difference in the total snoring ratio between the SnoreLab app and polygraphy was +11.7%, whereas the difference in summed-up loud and epic snoring recorded by SnoreLab was only −2.6% compared to the total snoring ratio of polygraphy (Figure 2). 

The Snore Score given by the app was on average 29.1 (SD 35.2), ranging from 1.0 to 117.0 (Table 2) and showed a very high correlation to the total snoring time measured by the app (r = 0.964). The snore index obtained from polygraphy had a mean value of 70.6 (SD 131.6), ranging from 0.0 to 450.1 (Table 2) and showed a lower correlation to the Snore Score of the app (r = 0.813). However, compared to the apnea-hypopnea-index (AHI) measured by polygraphy, all obtained snoring indices showed only a low to moderate correlation (r = 0.495–0.645, Table 4).

## 4. Discussion

To our knowledge, this is the first study which attempts to validate a commercially available smartphone application (SnoreLab) against polygraphy beyond a case report level. Even though current validation studies of commercially available snoring apps primarily included up to 13 different apps in their investigation, the clinical sample size was only one or two individuals [22,23]. Due to study-specific rating procedures, the SnoreLab app was never included [22,23]. Nevertheless, the app chosen by Camacho et al. [22] and validated against PSG revealed comparable sensitivities to the present investigation (64–96% versus 80–100%), although the positive prediction values were higher than those of the present study (93–96% versus 40–66.6%). Stippig et al. [23] investigated three snoring apps in comparison to PG, but did not report accuracy values. However, in concordance with the present results, the tendency for overrating snoring by the apps was also visible [23]. A third validation study of a paid snoring app [24] used neither PSG or PG, but analyzed the recordings of the app and compared the snoring rates measured by the app to those identified by an ear, nose and throat specialist: they revealed similar values as the present study regarding accuracy, positive prediction value, negative prediction value and specificity. However, direct comparison with the present study is limited not only to the different validation approach and the different app, but rather to the fact that the sample of Chiang et al. [24] was wearing an oral appliance which is reported to affect snoring and breathing and thus probably biased the reported values [24]. 

In contrast to commercial snoring apps, which are available at the common app stores, some research groups developed and tested their own smartphone apps for measurement of snoring and screening of OSA [25,26,35,36]. Focusing on those which solely use the smartphone without any supplementary external devices, two studies can be extrapolated [25,26]: Regarding the correlation between the snoring time measured by the smartphone compared to the respective gold standard, Nakano et al. [25] revealed a higher correlation than the present study (r = 0.93 versus r = 0.754/0.780). Moreover, both studies [25,26] found also higher correlations comparing the respiratory disturbance indices created by the apps with the AHI obtained by PSG/PG (r = 0.94 [25] versus r = 0.81 [26] versus r = 0.495). It has to be kept in mind, that the “Snore Score” calculated by SnoreLab is based on an unknown algorithm and thus cannot be directly compared to specific scores which are associated to respiratory events. A major limitation of commercially available apps is the limited access to raw data as well as the lack of transparency regarding the underlying measurement algorithms [17,37]. Therefore, one can argue if such specific scores generated by the app are suitable for research purposes. Regarding OSA screening, this has to be negated, because such scores are bearing too many major uncertainties. Moreover, the SnoreLab company states in their medical disclaimer, that “SnoreLab is not intended to diagnose or treat sleep apnea” [38]. In conjunction with the present results, it is neither intended nor appropriate for serving as a screening tool for OSA. Nevertheless, the present results revealed a high correlation between the Snore Score obtained by SL and the total snoring time recorded by SL. Thus, in patients seeking treatment of snoring, when OSA can be excluded by sufficient screening methods, the monitoring of such app indices could be useful in clinical practice for evaluation of treatment success. 

Beyond the free version of SnoreLab used for the present study, the SnoreLab company also offers a paid upgrade. While the free version records a limited number of audio samples per night, the paid version records the entire night. The use of the paid version in the present study could have had the advantage to enable an epoch-by-epoch comparison of snoring with PG and therefore a more detailed analysis of the SnoreLab measurements.

All smartphone snoring apps are audio-based and use the incorporated microphone. Given the large variability of mobile devices, differences caused by the different smartphone hardware components or the operating systems cannot be avoided [17,23,39]. For research purposes, one can argue that all participants should have used an identical smartphone, but regarding the commercial availability of the tested app, reliability of the measurements should be required. 

The present results could also have been affected by the fact that PG measures snoring via nasal pressure transducing instead of using a microphone. Interestingly, until now, no uniform standard method for the measurement of snoring has been determined. The American Association of Sleep Medicine (AASM) considers three methods to be equivalent: acoustic sensors, nasal pressure transducers and piezoelectric vibration sensors [40]. Taking this into account and considering the fact that PG is significantly more cost- and time-effective compared to PSG, the use of PG for validation studies seems acceptable [23,35]. 

As often criticized, one major limitation of commercial sleep and snore apps is the output of scores and statistics based on algorithms which are unknown to the research community as well as limited access to raw data [17,37], a fact which impedes the performance of evidence-based validation studies.

Further limitations of the current study have to be seen in the sample size comprising only 19 individuals. Despite the fact that comparable studies are rare and have smaller sample sizes, the validity of statistical analysis especially regarding the metrics obtained by means of the different snoring ratio thresholds (Table 3) is limited. The higher the arbitrary set threshold, the lower was the number of true positive values, which biased the calculation of the respective metrics. Furthermore, the post hoc power test considering the correlation between the total snoring ratios (r = 0.754) revealed a power of 55.1%, and was thus underpowered. The same was true for the correlation regarding the summed-up percentages of loud and epic snoring (r = 0.780), which resulted in a power of 65.2%. Given the effect size of the total snoring ratios, a sample size of 36 participants would have been needed to achieve a statistical power of 80%. In case of the summed-up percentages of loud and epic snoring, a sample size of 28 individuals would have been mandatory. Another limitation can be seen in the inequal distribution of male and female study participants. Due to the fact that snoring has a male predominance among the general population [1,2], the present distribution could have affected the results slightly. Due to the audio-based approach of all smartphone snoring apps, background noises have a great impact on data collection and recording. While background noises caused by bedpartners, pets or open windows had been eliminated during the respective study night, they probably cannot be ruled out if an app-based approach should be used clinically for monitoring the success of treatment against snoring. 

Overall, some questions regarding snoring apps in general and particularly the SnoreLab app still remain open. Further research is needed to validate snoring apps as the present one in a larger patient sample, which is more homogenous regarding gender distribution and presence/absence of OSA. An epoch-by-epoch comparison between app and PG could provide more detailed information of the SnoreLab measurements and therefore overcome the limitation of unknown underlying algorithms to a certain extent.

## 5. Conclusions

An overestimation of snoring by the app was seen. The best agreement between both methods was achieved in comparing the summed-up ratios of loud and epic snoring obtained by SnoreLab with the total snoring ratio measured by PG. Compared to PG, the SnoreLab app only provided acceptable accuracy values for measuring snoring >50% per night: an accuracy of 94.7%, a sensitivity of 100%, a specificity of 94.1%, a positive prediction value of 66.6% and a negative prediction value of 100%. Using the extractable data of the app, no association to respiratory indices obtained by PG could be found. 

For clinical practice, the present results indicate that the SnoreLab app should not be used for OSA screening, but can possibly be a helpful tool for monitoring snoring, e.g., in conjunction with the evaluation of different treatment modalities.

## Figures and Tables

**Table 1:** Cross table for calculation of sensitivity, specificity, positive and negative prediction values, prevalence and accuracy.

		Polygraphy (Gold Standard)	
		Snoring beyond threshold	Snoring below threshold
**SnoreLab app**	Snoring beyond threshold	True positive (TP)	False positive (FP)
	Snoring below threshold	False negative (FN)	True negative (TN)

**Table 2:** Median, minimal and maximal values as well as means and standard deviations of measurements derived by the SnoreLab app and polygraphy.

	Median	Min	Max	Mean	SD
**SnoreLab app**					
Monitoring time (hh:mm:ss)	07:23:00	05:13:00	11:44:00	07:27:44	01:21:30
Total snoring (%)	26.3	0.2	72.8	25.3	22.0
Light snoring (%)	9.7	0.2	39.8	14.3	12.9
Loud snoring (%)	3.5	0.0	31.6	7.8	9.9
Epic snoring (%)	0.2	0.0	22.1	3.2	6.5
Loud + epic snoring (%)	5.4	0.0	53.6	10.9	15.8
Snore Score	15.0	1.0	117.0	29.1	35.2
**Polygraphy**					
Monitoring time (hh:mm:ss)	07:29:58	05:16:02	10:00:00	07:26:57	01:00:58
Total snoring (%)	2.4	0.0	68.4	13.1	21.5
Snore index	7.9	0.0	450.1	70.6	131.6
Snore index obstructive	0.1	0.0	38.4	4.4	9.6
Apnea-hypopnea-index	9.7	0.9	38.7	12.2	10.4

Abbreviations: Min = minimum, Max = maximum, SD = standard deviation, h = hours, m = minutes, s = seconds.

**Table 3:** Prevalence, sensitivity, specificity, positive predictive value, negative predictive value and accuracy of snoring detection by the SnoreLab app relative to polygraphy (used as gold standard). Snoring was classified as present if the different threshold levels of total snoring per night were exceeded. Values are given in percentage (%).

	Thresholds of Percentage of Total Snoring per Night			
	>50%	>25%	>10%	>5%
Prevalence	10.5	21.1	31.5	42.1
Sensitivity	100.0	80.0	100.0	100.0
Specificity	94.1	57.1	53.8	54.5
Positive predictive value	66.6	40.0	50.0	61.5
Negative predictive value	100.0	88.8	100.0	100.0
Accuracy	94.7	63.2	68.4	73.6

**Table 4:** Spearman-Rho correlation coefficients (r) for the different scores obtained by the SnoreLab app (SL) and polygraphy (PG).

Variables	r
Snore Score (SL)/Snore index (PG)	0.813
Snore index (PG)/Snore index obstructive (PG)	0.809
AHI (PG)/Snore Score (SL)	0.495
AHI (PG)/Snore index (PG)	0.576
AHI (PG)/Snore index obstructive (PG)	0.645

## Data Availability

The data presented in this study are available on reasonable request from the corresponding author.

## References

[1] Chan C.H., Wong B.M., Tang J.L., Ng D.K. (2012). Gender difference in snoring and how it changes with age: Systematic review and meta-regression. Sleep Breath..

[2] Li C., Hoffstein V., Kryger M.H., Roth T., Dement W.C. (2011). Snoring. Principles and Practice of Sleep Medicine.

[3] American Academy of Sleep Medicine (2014). International Classification of Sleep Disorders.

[4] Myers K.A., Mrkobrada M., Simel D.L. (2013). Does this patient have obstructive sleep apnea? The Rational Clinical Examination systematic review. JAMA.

[5] Patel S.J. (2019). Obstructive Sleep Apnea. Ann. Intern. Med..

[6] Jordan A.S., McSharry D.G., Malhotra A. (2014). Adult obstructive sleep apnoea. Lancet.

[7] Semelka M., Wilson J., Floyd R. (2016). Diagnosis and treatment of obstructive sleep apnea in adults. Am. Fam. Physician.

[8] Bradley T.D., Floras J.S. (2009). Obstructive sleep apnoea and its cardiovascular consequences. Lancet.

[9] Hillman D., Mitchell S., Streatfield J., Burns C., Bruck D., Pezzullo L. (2018). The economic cost of inadequate sleep. Sleep.

[10] Kapur V., Strohl K.P., Redline S., Iber C., O’Connor G., Nieto J. (2002). Underdiagnosis of sleep apnea syndrome in U.S. communities. Sleep Breath..

[11] Mold J.W., Quattlebaum C., Schinnerer E., Boekman L., Orr W., Hollabaugh K. (2011). Identification by primary care clinicians of patients with obstructive sleep apnea: A practice-based research network (PBRN) study. J. Am. Board Fam. Med..

[12] Stuck B.A., Braumann B., Heiser C., Herzog M., Maurer J.T., Plößl S., Steffen A., Sommer J.U., Verse T., Hofauer B. (2019). S3-Leitlinie “Diagnostik und Therapie des Schnarchens des Erwachsenen”. Somnologie.

[13] Mayer G., Arzt M., Braumann B., Ficker J.H., Fietze I., Galetke W., Maurer T., Orth M., Penzel T., Pistner H.P. (2017). S3-Leitlinie Nicht erholsamer Schlaf/Schlafstörungen–Kapitel “Schlafbezogene Atmungsstörungen”. Somnologie.

[14] Stuck B.A., Arzt M., Fietze I., Galetke W., Hein H., Heiser C., Herkenrath S.D., Hofauer B., Maurer J.T., Mayer G. (2020). Teil-Aktualisierung S3-Leitlinie Schlafbezogene Atmungsstörungen bei Erwachsenen. Somnologie.

[15] Kusheida C.A., Litter M.R., Morgenthaler T., Alessi C.A., Bailey D., Coleman J., Friedman L., Hirshkowitz M., Kapen S., Kramer M. (2005). Practice parameters for the indications for polysomnography and related procedures. An update for 2005. Sleep.

[16] Kapur V.K., Auckley D.H., Chowdhuri S., Kuhlmann D.C., Mehra R., Ramar K., Harrod C.G. (2017). Clinical practice guideline for diagnostic testing for adult obstructive sleep apnea: An American Academy of Sleep Medicine clinical practice guideline. J. Clin. Sleep Med..

[17] Behar J., Roebuck A., Domingos J.A., Gederi E., Clifford G.D. (2013). A review of current sleep screening applications for smartphones. Physiol. Meas..

[18] Lindemann J., Augenstein B., Stupp F., Saul B., Reichert M., Riepl R., Sommer F., Grossi A.S. (2017). Diagnostische Genauigkeit ambulanter Polygrafiegeräte. HNO.

[19] https://research2guidance.com/mhealth-app-developer-economics/.

[20] Baron K.G., Duffecy J., Berendsen M.A., Cheung I.N., Lattie E., Manalo N.C. (2018). Feeling validated yet? A scoping review of the use of consumer-targeted wearable and mobile technology to measure and improve sleep. Sleep Med. Rev..

[21] Fino E., Mazzetti M. (2019). Monitoring healthy and disturbed sleep through smartphone applications: A review of experimental evidence. Sleep Breath..

[22] Camacho M., Robertson M., Abdullatif J., Certal V., Kram Y.A., Ruoff C.M., Brietzke S.E., Capasso R. (2015). Smartphone apps for snoring. J. Laryngol. Otol..

[23] Stippig A., Hübers U., Emerich M. (2015). Apps in sleep medicine. Sleep Breath..

[24] Chiang J.K., Lin Y.C., Lin C.W., Ting C.S., Chiang Y.Y., Kao Y.H. (2021). Validation of snoring detection using a smartphone app. Sleep Breath..

[25] Nakano H., Hirayama K., Sadamitsu Y., Toshimitsu A., Fujita H., Hin S., Tanigawa T. (2014). Monitoring sound to quantify snoring and sleep apnea severity using a smartphone: Proof of concept. J. Clin. Sleep Med..

[26] Tiron R., Lyon G., Kilroy H., Osman A., Kelly N., O’Mahony N., Lopes C., Coffey S., McMahon S., Wren M. (2020). Screening for obstructive sleep apnea with novel hybrid acoustic smartphone app technology. J. Thorac. Dis..

[27] The Federal Health Monitoring System, Germany. https://www.gbe-bund.de/gbe/pkg_isgbe5.prc_menu_olap?p_uid=gast&p_aid=52123572&p_sprache=D&p_help=2&p_indnr=434&p_version=2&p_ansnr=72674692.

[28] Field A. (2013). Discovering Statistics Using IBM SPSS Statistics.

[29] Shapiro S.S., Wilk M.B. (1965). Analysis of variance test for normality (complete samples). Biometrika.

[30] Hinkle D.E., Wiersma W., Jurs S.G. (2003). Applied Statistics for the Behavioral Sciences.

[31] Altman D.G., Bland J.M. (1983). Measurement in medicine: The analysis of method comparison studies. Statistician.

[32] Bland J.M., Altman D.G. (1986). Statistical methods for assessing agreement between two methods of clinical measurement. Lancet.

[33] Faul F., Erdfelder E., Lang A.G., Buchner A. (2007). G*Power 3: A flexible statistical power analysis program for the social, behavioral, and biomedical sciences. Behav. Res. Methods.

[34] Faul F., Erdfelder E., Buchner A., Lang A.G. (2009). Statistical power analysis using G*Power 3.1: Test for correlation and regression analyses. Behav. Res. Methods.

[35] Behar J., Roebuck A., Shadid M., Daly J., Hallack A., Palmuis N., Stradling J., Clifford G.D. (2015). SleepAp: An automated obstructive sleep apnoea screening application for smartphones. IEEE J. Biomed. Health Inform..

[36] Burgdorf A., Güthe I., Jovanovic M., Kutafina E., Kohlschein C., Bitsch J.A., Jonas S.M. (2018). The mobile sleep lab app: An open-source framework for mobile sleep assessment based on consumer-grad wearable devices. Comput. Biol. Med..

[37] Lorenz C.P., Williams A.J. (2017). Sleep apps: What role do they play in clinical medicine?. Curr. Opin. Pulm. Med..

[38] SnoreLab-Homepage. https://www.snorelab.com/faqs/.

[39] Figueras-Alvarez O., Cantó-Navés O., Cabratosa-Termes J., Roig-Cayón M., Felipe-Spada N., Tomàs-Aliberas J. (2020). Snoring intensity assessment with three different smartphones using the SnoreLab application in one participant. J. Clin. Sleep Med..

[40] Berry R.B., Brooks R., Gamaldo C.E., Harding S.M., Lloyd R.M., Marcus C.L., Vaugh B.V. (2015). The AASM Manual for the Scoring of Sleep and Associated Events: Rules, Terminology and Technical Specifications.

